# Crystal structure of a three-coordinate lithium complex with monodentate phenyl­oxazoline and hexa­methyl­disilyl­amide ligands

**DOI:** 10.1107/S2056989024004237

**Published:** 2024-05-17

**Authors:** José Severiano Carneiro Neto, Eduardo Mariano Iwaya, Francielli Sousa Santana, Jaísa Fernandes Soares

**Affiliations:** a Departamento de Química, Universidade Federal do Paraná, Centro Politécnico, Jardim das Américas, 81530-900, Curitiba-PR, Brazil; Illinois State University, USA

**Keywords:** 4,4-dimethyl-2-phenyl-2-oxazoline, trigonal planar, lithium(I), hexa­methyl­disilyl­amide, crystal structure

## Abstract

The reaction of lithium hexa­methyl­disilyl­amide, [Li{N(Si(CH_3_)_3_)_2_}] (LiHMDS), with 4,4-dimethyl-2-phenyl-2-oxazoline (Phox, C_11_H_13_NO) in hexane produced colourless crystals of bis­(4,4-dimethyl-2-phenyl-2-oxazoline-κ*N*)(hexa­methyl­disilyl­amido-κ*N*)lithium, [Li(C_6_H_18_NSi_2_)(C_11_H_13_NO)_2_] or [Li{N(Si(CH_3_)_3_)_2_}(Phox)_2_] in high yield (89%). Despite the 1:1 proportion of the starting materials in the reaction mixture, the product formed with a 1:2 amide:oxazoline ratio. In the unit cell of the *C*2/*c* space group, the neutral mol­ecules lie on twofold rotation axes coinciding with the Li—N(amide) bonds. The lithium(I) centre adopts a trigonal–planar coordination geometry with three nitro­gen donor atoms, one from the HMDS anion and two from the oxazolines. All ligands are monodentate. In the phenyl­oxazoline units, the dihedral angle defined by the five-membered heterocyclic rings is 35.81 (5)°, while the phenyl substituents are approximately face-to-face, separated by 3.908 (5) Å. In the amide, the methyl groups assume a nearly eclipsed arrangement to minimize steric repulsion with the analogous substituents on the oxazoline rings. The non-covalent inter­actions in the solid-state structure of [Li{N(Si(CH_3_)_3_)_2_}(Phox)_2_] were assessed by Hirshfeld surface analysis and fingerprint plots. This new compound is attractive for catalysis due to its unique structural features.

## Abstract 2

The reaction between lithium hexa­methyl­disilazane, [Li{N(Si(CH_3_)_3_)_2_}] (LiHMDS), with 4,4-dimethyl-2-phenyl-2-oxazoline (Phox) in hexane produced the title complex [Li{N(Si(CH_3_)_3_)_2_}(Phox)_2_]. The mol­ecule, which crystallizes in the *C*2/*c* space group, lies on a twofold rotation axis and the lithium cation adopts a trigonal–planar coordination environment by the coordination, through nitro­gen atoms, of one unit of hexa­methyl­disilazane and two units of Phox, both ligands in a monodentate mode.

## 1. Chemical context

Oxazolines are a family of cyclic amino­ethers characterized by five-membered heterocyclic rings containing one unsaturation. They can be prepared using various methods that typically involve the cyclization of an amino­alcohol as a general process (Mulahmetovic & Hargaden, 2019 ▸). These compounds have been widely used in synthesis, catalysis, and as proligands in coordination chemistry (Connon *et al.*, 2021 ▸; Liu *et al.*, 2016 ▸; Rasappan *et al.*, 2008 ▸; McManus & Guiry, 2004 ▸; Gómez *et al.*, 1999 ▸).

Metal complexes containing monodentate, *N*-donor monooxazoline ligands are reasonably frequent in the solid state (Huang *et al.*, 2014 ▸; Del Río & Gossage, 2009 ▸; Barclay *et al.*, 2003 ▸; Valk *et al.*, 1994 ▸; Michelin *et al.*, 1991 ▸). In the case of 2-phenyl­oxazolines (Phox), which are of inter­est to this work, the chelating behaviour is more common and involves a second donor atom, generally N, O, S or Se, in the *ortho* position of the aromatic ring (Volpe *et al.*, 2010 ▸; Bottini *et al.*, 2010 ▸). Phox complexes of *d-*block metals have been studied as catalysts (Abu-Elfotoh, 2017 ▸; Bagherzadeh *et al.*, 2008 ▸; Kandasamy *et al.*, 2004 ▸).

Lithium hexa­methyl­disilyl­amide (LiHMDS), in turn, was first crystallographically characterized as a cyclic, trimeric compound with alternating nitro­gen and lithium atoms in a planar six-membered ring (Mootz *et al.*, 1969 ▸). This complex and other bulky *M*N(Si*R*_3_)_2_ bis­(tri­alkyl­sil­yl)amides (*M* = alkali metal; *R* = Me, Et, ^*i*^Pr, ^*t*^Bu, Ph, *etc*.) are widely used in organic synthesis as deprotonating agents due to their low nucleophilicity and strong Brønsted basicity (Neufeld *et al.*, 2016 ▸; Tang *et al.*, 2005 ▸; Beak *et al.*, 1996 ▸), and in coordination chemistry as sterically demanding starting materials to impose low coordination numbers (Mohamed, 2010 ▸; Power, 2004 ▸). In both fields, the high solubility conferred by the tri­methyl­silyl substituents in a wide range of non-polar organic solvents is an advantage of working with HMDS^−^ in synthetic procedures (Ojeda-Amador *et al.*, 2016 ▸).

In this paper, we report the synthesis, crystal and mol­ecular structures of the mononuclear, three-coordinate lithium(I) complex [Li{N(Si(CH_3_)_3_)_2_}(Phox)_2_] (Phox = 4,4-dimethyl-2-phenyl-2-oxazoline). The product crystallized directly from the reaction mixture at 253 K, following an attempt to deprotonate the oxazoline by reaction between [LiN(Si(CH_3_)_3_)_2_] and Phox in hexane. 
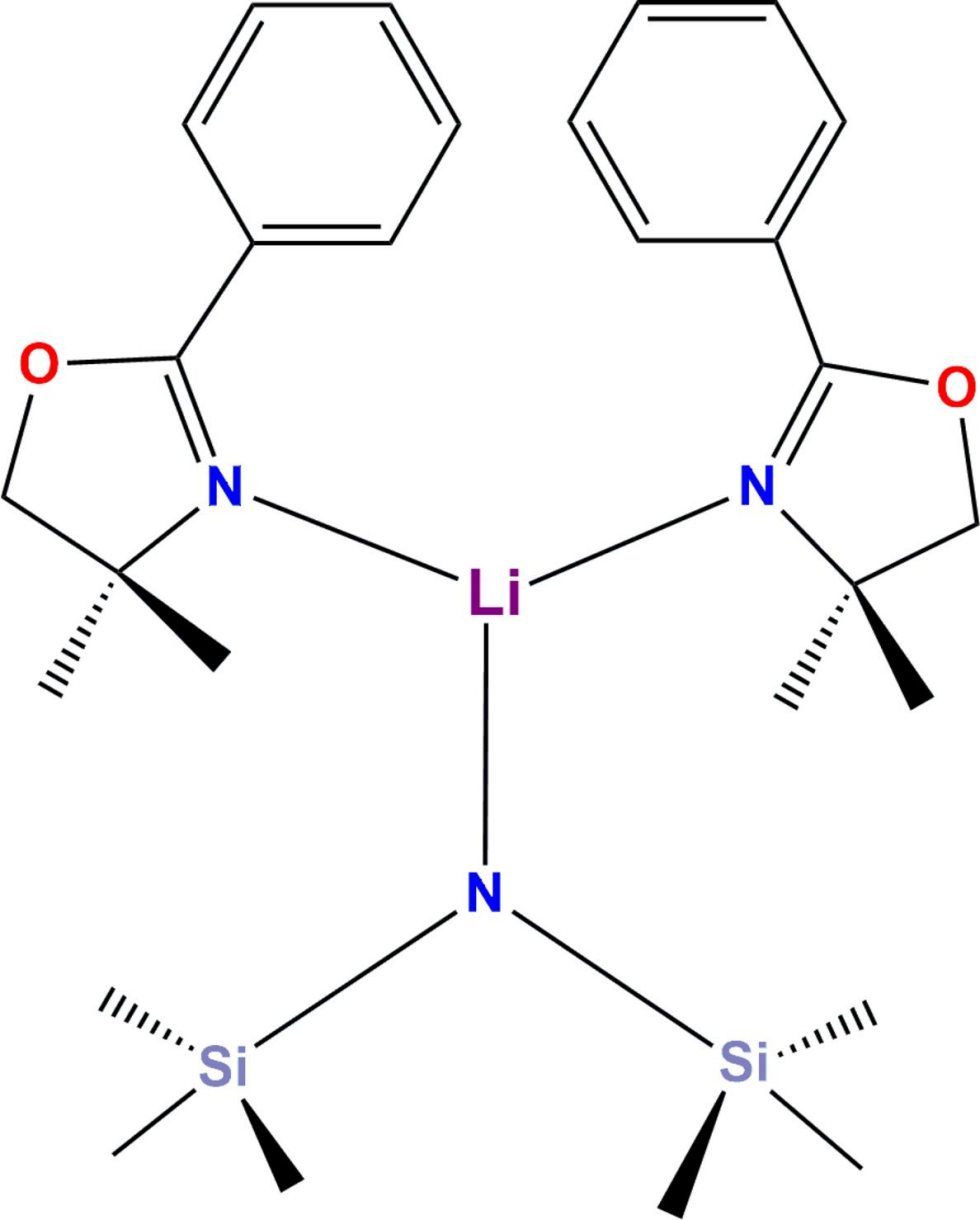


## 2. Structural commentary

**Figure 1:**
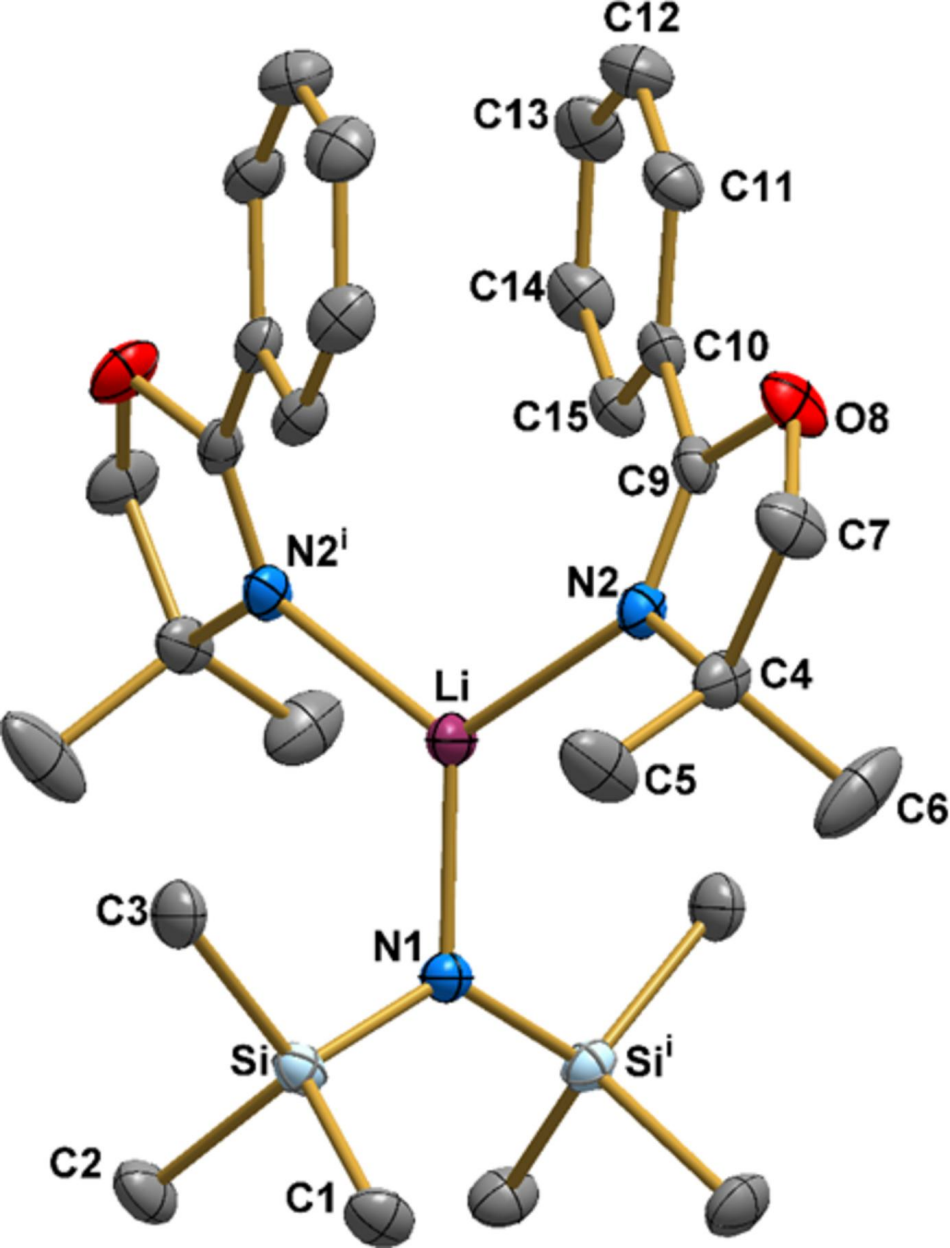
View of the mol­ecular structure of [Li{N(Si(CH_3_)_3_)_2_}(Phox)_2_], with the atom-numbering scheme. Hydrogen atoms are omitted for clarity. Displacement ellipsoids are drawn at the 50% probability level. Symmetry code: (i) −*x* + 1, *y*, −*z* + 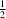 .

**Figure 2:**
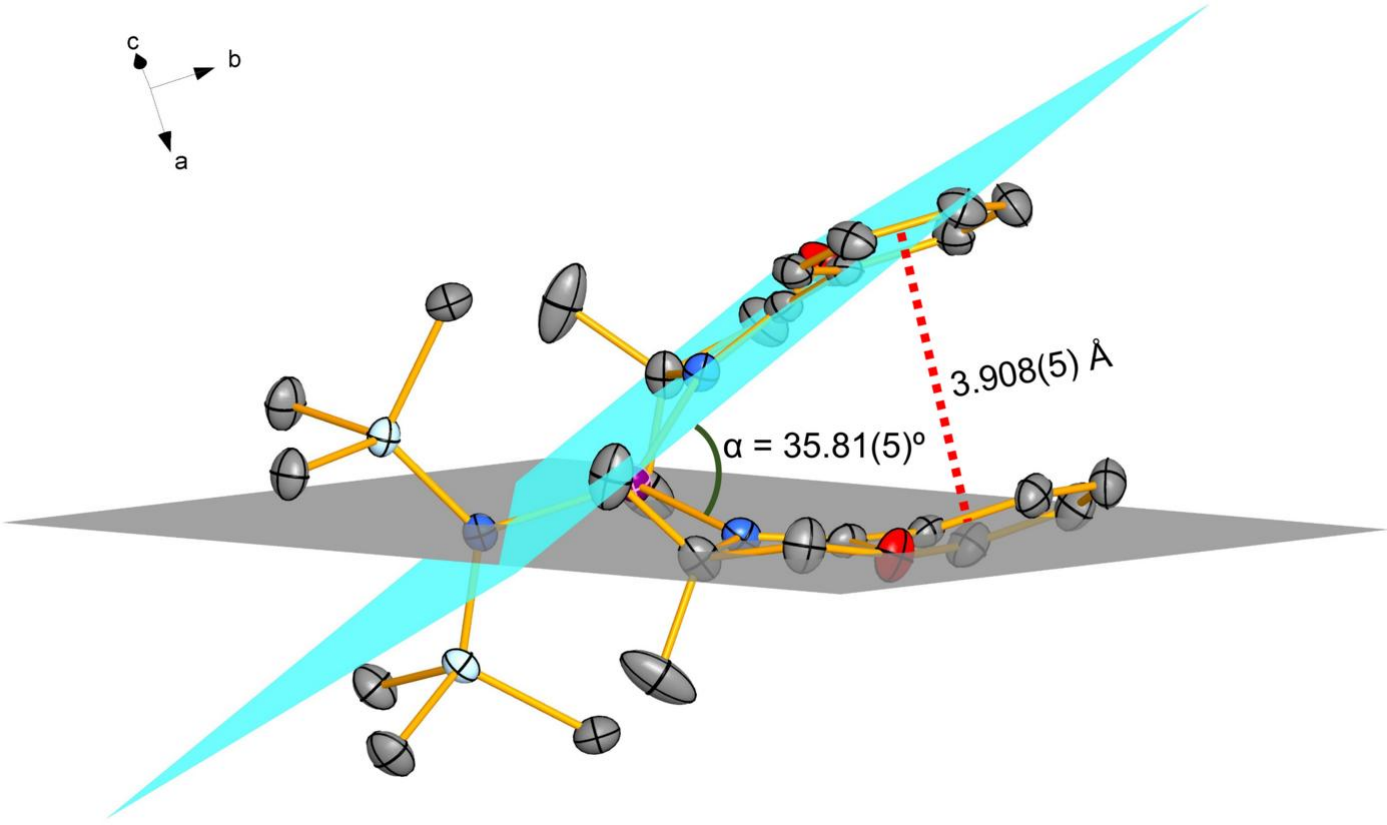
Distance between the phenyl rings (red dashed line) and dihedral angle (α) between the five-membered oxazoline rings. Grey: carbon; red: oxygen; blue: nitro­gen; light blue: silicon; purple: lithium. Displacement ellipsoids are drawn at the 50% probability level. Hydrogen atoms are excluded for clarity.

The [Li{N(Si(CH_3_)_3_)_2_}(Phox)_2_] complex crystallizes in the *C*2/*c* space group as a discrete mol­ecular unit with no solvent in the unit cell. The asymmetric unit comprises half of the anionic hexa­methyl­disilyl­amide ligand, {N_0.5_(Si(CH_3_)_3_)}^0.5–^, and one neutral 4,4-dimethyl-2-phenyl-2-oxazoline mol­ecule, both coordinated to the lithium centre solely by the nitro­gen atoms. Proper rotation about a twofold axis passing through the Li—N1 bond (symmetry operation −*x* + 1, *y*, −*z* + 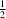 ) leads to the complete, neutral lithium complex mol­ecule. The coordination sphere of lithium(I) presents a trigonal–planar geometry with no deviation from planarity, Fig. 1 ▸. In this environment, the two five-membered oxazoline rings (each with a planarity deviation of 0.082°) form a dihedral angle (α) of 35.81 (5)°. At the same time, the distance between the approximately face-to-face phenyl substituents equals 3.908 (5) Å, Fig. 2 ▸.

The Li—N bonds are 2.126 (2) and 1.942 (3) Å long for the oxazoline and amide ligands, respectively. Such a significant bond-length variation comes from the stronger inter­action of the lithium centre with the anionic amide nitro­gen (higher negative density charge) compared to the neutral oxazoline. Similar 0.1–0.2 Å bond-distance differences have been used to distinguish ‘ionic’ from ‘dative’ Li—N bonds (Henderson *et al.*, 1997 ▸; Gregory *et al.*, 1991 ▸) and were reported earlier for other mononuclear, trigonal planar Li complexes containing an amide (N{Si*R*_3_}_2_^−^, NArH^−^) and a neutral ligand such as sparteine, *N,N,N′,N′*-tetra­methyl­ethylenedi­amine, and *N′,N′,N′′,N′′,N′′′*-tetra­methyl­ethylenedi­amine (Clark *et al.*, 2009 ▸; Henderson *et al.*, 1997 ▸; Fjeldberg *et al.*, 1984 ▸). The Li—N_oxazoline_ bond is longer than analogous bonds reported for dimeric lithium(I) complexes with bidentate [Jantzi *et al.*, 2006 ▸; average 2.044 (3) Å] and tridentate oxazoline ligands [Stol *et al.*, 2005 ▸; average 1.996 (3) Å]; the difference can be ascribed to distinct Li^+^ coordination numbers and ligands’ denticities and bulk. To our knowledge, the title compound is the first example of a monodentate oxazoline complex of lithium(I) structurally characterized in the solid state.

**Figure 3:**
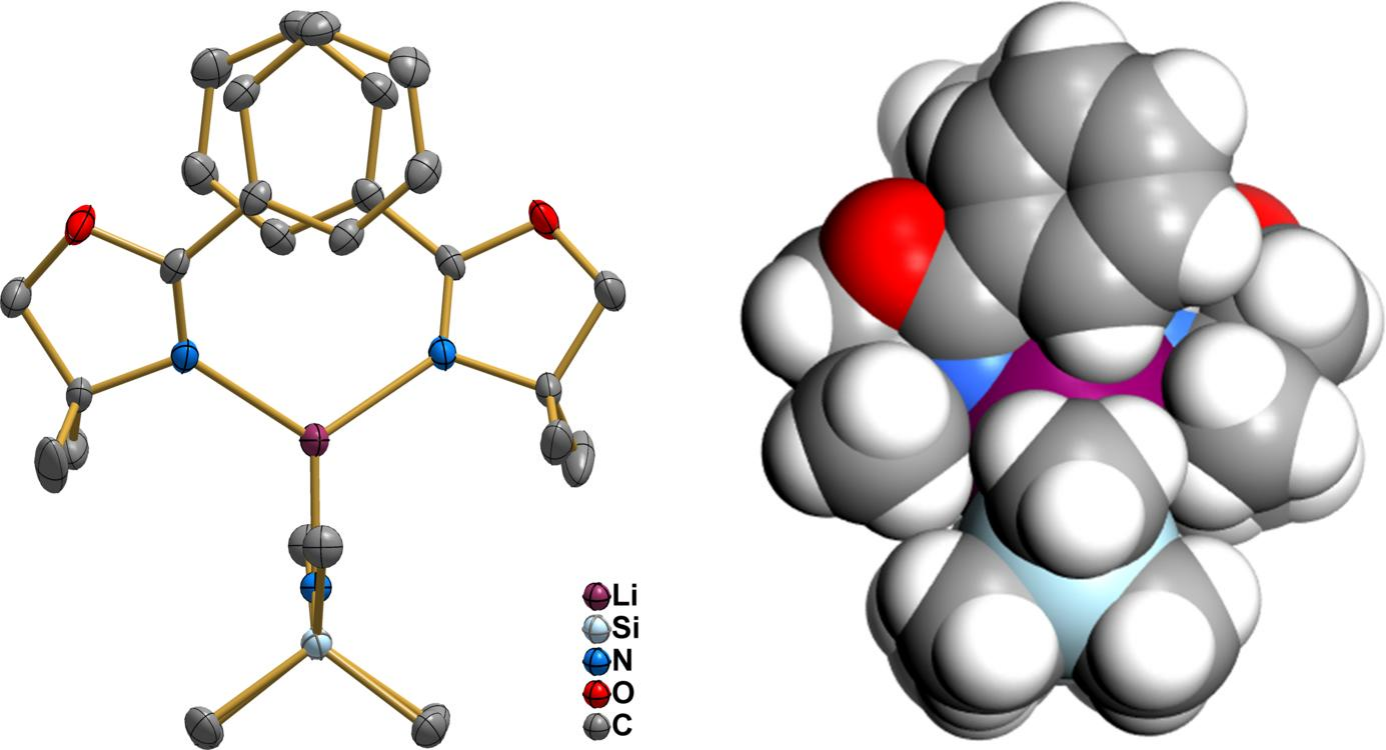
Left: Mol­ecular structure of [Li{N(Si(CH_3_)_3_)_2_}(Phox)_2_], showing the eclipsed conformation of the hexa­methyl­disilyl­amide ligands. Hydrogen atoms are omitted for clarity. Displacement ellipsoids are drawn at the 50% probability level. Right: Space-filling representation of the title compound, emphasizing the efficient shielding of the lithium cation by the methyl and phenyl substituents of the HMDS^−^ and Phox ligands.

In [Li{N(Si(CH_3_)_3_)_2_}(Phox)_2_], the methyl groups of the hexa­methyl­disilyl­amide moieties are nearly eclipsed (Fig. 3 ▸), with a dihedral angle of 5.80 (7)° between the C3—Si—N1 and the C3^i^—Si^i^—N1 planes. Additionally, if one defines a vector connecting the symmetry-related Si and Si^i^ centres (Randazzo *et al.*, 2006 ▸), the relative positions of the C3/C3^i^, C2/C1^i^ and C1/C2^i^ pairs across this vector produce C—Si⋯Si^i^—C^i^ torsion angles of −8.846 (11), −2.52 (7) and −2.52 (7)°, respectively (average value −4.63°). These figures are close to the 0° value attributed to the eclipsed conformation (Eliel & Wilen, 1994 ▸). Small torsion angles also appear in the amide ligand of [Cs{N(SiMe_3_)_2_}(tmeea)] (tmeea = tris­[2-(2-meth­oxy­eth­oxy)eth­yl]amine)], [{K(N{SiMe_3_}_2_)(^*t*^Bu-C_6_H_5_)}_2_], and [{K(N{SiMe_3_}_2_)(Me_3_C_6_H_3_)}_2_], with average values of −3.81 (4), 4.70 and 0.75° respectively (Ojeda-Amador *et al.*, 2016 ▸; Randazzo *et al.*, 2006 ▸). A larger deviation from 0° appears in [K(18-crown-6)][Li{N(SiMe_3_)_2_}_2_] (mean torsion angle −11.40°; Davison *et al.*, 2023 ▸) and in the polymeric [{(Me_3_Si)_2_NLi}{(Me_3_Si)_2_NK}]_∞_ (–11.94°; Morris *et al.*, 2007 ▸). On the other hand, the methyl groups of unsolvated K(N{SiMe_3_}_2_) adopt an inter­mediate mean torsion angle of 38.29° (Tesh *et al.*, 1990 ▸), while in [Li{N(SiMe_3_)_2_}(Me_6_Tren)] (Me_6_Tren = tris­[2-(di­methyl­amino)­eth­yl]amine) the average angle is 59.9°, almost exactly the ideal value of 60° for a staggered conformation (Cousins *et al.*, 2010 ▸). In the present work, eclipsing implies less repulsion between the bulky methyl groups of both the hexa­methyl­disilyl­amide and Phox ligands than in the staggered arrangement.

## 3. Supra­molecular features

**Figure 4:**
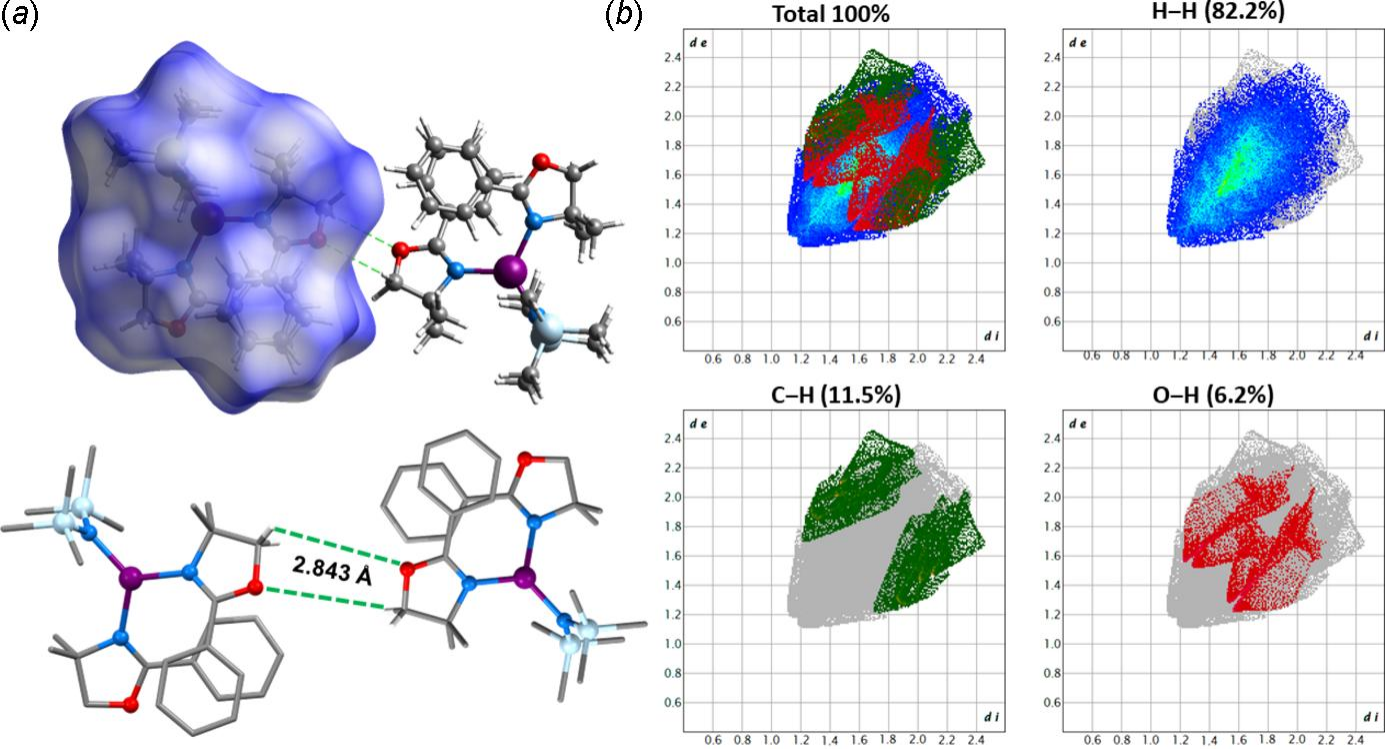
(*a*) Representation of the Hirshfeld surface for [Li{N(Si(CH_3_)_3_)_2_}(Phox)_2_], highlighting the inter­molecular O⋯H contacts (green dashed lines) between the oxazoline’s methyl­ene moiety and the oxygen atom of the adjacent mol­ecule. Grey: carbon; red: oxygen; blue: nitro­gen; light blue: silicon; purple: lithium. Hydrogen atoms are omitted for clarity. (*b*) Two-dimensional fingerprint plots in the *d_norm_* function, generated by mapping, for each *d_i_*, the region between 0.4 and 2.6 Å from the surface to the nearest inter­nal (*d_i_*) and external (*d_e_*) atoms.

Hirshfeld surface analysis performed with the *CrystalExplorer 21.5* software (Spackman *et al.*, 2021 ▸) allowed a comprehensive examination of the non-covalent bonds governing the solid-state structure of [Li{N(Si(CH_3_)_3_)_2_}(Phox)_2_]. A pivotal aspect of this analysis involves the generation of 2D fingerprint plots (FP), which offer two-dimensional projections of the Hirshfeld surface (Hirshfeld, 1977 ▸). This enables meticulous analysis of the non-covalent forces supporting the supra­molecular structure by qu­anti­fying the percentage contribution of each inter­action. For [Li{N(Si(CH_3_)_3_)_2_}(Phox)_2_], the percentages of the total surface area corres­ponding to the H⋯H, C⋯H, and O⋯H contacts account for 82.2%, 11.5%, and 6.2%, respectively (Fig. 4 ▸). The frail O⋯H contacts occur between the hydrogen atoms of the methyl­ene groups in the oxazoline rings and the oxygen atom of the adjacent mol­ecules at 2.843 Å. Besides those, there is only a weak intra­molecular π–π stacking inter­action between the aromatic rings of the Phox ligands, as already depicted in Fig. 2 ▸, which are 3.908 (5) Å far from one another. There is no classic intra or inter­molecular hydrogen bonding in the mol­ecule.

## 4. Database survey

To the best of our knowledge, this is the first example of a heteroleptic lithium(I)–oxazoline complex in which the Phox ligands are monodentate and the HMDS anion adopts a nearly eclipsed conformation of the methyl groups, giving rise to a trigonal planar coordination environment about the metal. The combination of ten methyl and two phenyl substituents in the ligands efficiently shields the central ion (Fig. 3 ▸, right) and prevents significant inter­molecular contacts involving the metal.

## 5. Synthesis and crystallization

The reactions were carried out under di­nitro­gen (99.999%, Praxair or Air Liquide) using Schlenk techniques. Analytical grade 2-amino-2-methyl-1-propanol, ethyl­eneglycol, potassium carbonate, glycerol, benzo­nitrile, *n*-butyl lithium (2.5 mol L^−1^ solution in hexa­nes) and hexa­methyl­disilazane were acquired from commercial sources (Sigma-Aldrich, Merck, Synth) and used without purification. Hexane (Honeywell) was dried by standard methods (Armarego & Perrin, 1997 ▸) and distilled under N_2_(g) immediately before use. Lithium hexa­methyl­disilyl­amide, LiHMDS (Tai *et al.*, 2017 ▸), and Phox (Mulahmetovic & Hargaden, 2019 ▸) were prepared using procedures adapted from the literature; Phox was distilled twice under vacuum before use. A solution of 0.472 g (2.85 mmol) of LiHMDS in 10 mL of hexane was added slowly to a hexane solution of 0.500 g (2.85 mmol) of Phox at 273 K. The colourless reaction mixture was stirred at room temperature for about 5 h and filtered through Celite. The resulting colourless solution was cooled down to 253 K for two days, after which block-shaped colourless crystals were isolated by filtration and dried under vacuum. Total yield: 0.621g (85.0%) based on the [Li{N(Si(CH_3_)_3_)_2_}(Phox)_2_] formulation.

## 6. Refinement

Table 1 ▸ summarizes crystal data, data collection, and structure refinement details. The hydrogen atoms were located in difference-Fourier maps and were refined freely, except for the hydrogen atoms attached to C1, C6, C7, C11, C14, and C15 atoms, for which distance restraints (DFIX) were applied.

## Supplementary Material

Crystal structure: contains datablock(s) I. DOI: 10.1107/S2056989024004237/ej2003sup1.cif

Structure factors: contains datablock(s) I. DOI: 10.1107/S2056989024004237/ej2003Isup2.hkl

Supporting information file. DOI: 10.1107/S2056989024004237/ej2003Isup3.mol

CCDC reference: 2354028

Additional supporting information:  crystallographic information; 3D view; checkCIF report

## Figures and Tables

**Table 1:** Experimental details

Crystal data	
Chemical formula	[Li(C_6_H_18_NSi_2_)(C_11_H_13_NO)_2_]
*M*_r_	517.78
Crystal system, space group	Monoclinic, *C*2/*c*
Temperature (K)	100
*a*, *b*, *c* (Å)	15.612 (2), 12.7649 (17), 17.180 (4)
β (°)	116.243 (5)
*V* (Å^3^)	3070.9 (9)
*Z*	4
Radiation type	Mo *K*α
μ (mm^−1^)	0.14
Crystal size (mm)	0.50 × 0.44 × 0.28

Data collection	
Diffractometer	Bruker D8 Venture/Photon 100 CMOS
Absorption correction	Multi-scan (*SADABS*; Krause *et al.*, 2015 ▸)
*T*_min_, *T*_max_	0.709, 0.746
No. of measured, independent and observed [*I* > 2σ(*I*)] reflections	44358, 3522, 2888
*R*_int_	0.078
(sin θ/λ)_max_ (Å^−1^)	0.650

Refinement	
*R*[*F* ^2^ > 2σ(*F* ^2^)], *wR*(*F* ^2^), *S*	0.035, 0.092, 1.04
No. of reflections	3522
No. of parameters	252
No. of restraints	7
H-atom treatment	All H-atom parameters refined
Δρ_max_, Δρ_min_ (e Å^−3^)	0.29, −0.24

Computer programs: *APEX3* and *SAINT* (Bruker, 2015 ▸), *SHELXS2008* (Sheldrick 2008 ▸), *SHELXL2018/3* (Sheldrick, 2015 ▸), *DIAMOND* (Brandenburg & Putz, 1999 ▸), and *Mercury* (Macrae *et al.*, 2020 ▸) and *WinGX* (Farrugia, 2012 ▸).

## supplementary crystallographic information

### Crystal data

**Table:**

[Li(C_6_H_18_NSi_2_)(C_11_H_13_NO)_2_]	*F*(000) = 1120
*M**_r_* = 517.78	*D*_x_ = 1.120 Mg m^−^^3^
Monoclinic, *C*2/*c*	Mo *K*α radiation, λ = 0.71073 Å
*a* = 15.612 (2) Å	Cell parameters from 9967 reflections
*b* = 12.7649 (17) Å	θ = 2.9–27.7°
*c* = 17.180 (4) Å	µ = 0.14 mm^−^^1^
β = 116.243 (5)°	*T* = 100 K
*V* = 3070.9 (9) Å^3^	Parallelepiped, colourless
*Z* = 4	0.50 × 0.44 × 0.28 mm

### Data collection

**Table:**

Bruker D8 Venture/Photon 100 CMOS diffractometer	3522 independent reflections
Radiation source: fine-focus sealed tube	2888 reflections with *I* > 2σ(*I*)
Graphite monochromator	*R*_int_ = 0.078
Detector resolution: 10.4167 pixels mm^-1^	θ_max_ = 27.5°, θ_min_ = 2.9°
φ and ω scans	*h* = −20→20
Absorption correction: multi-scan (SADABS; Krause *et al.*, 2015)	*k* = −16→16
*T*_min_ = 0.709, *T*_max_ = 0.746	*l* = −22→22
44358 measured reflections	

### Refinement

**Table:**

Refinement on *F*^2^	Primary atom site location: structure-invariant direct methods
Least-squares matrix: full	Secondary atom site location: difference Fourier map
*R*[*F*^2^ > 2σ(*F*^2^)] = 0.035	Hydrogen site location: difference Fourier map
*wR*(*F*^2^) = 0.092	All H-atom parameters refined
*S* = 1.04	*w* = 1/[σ^2^(*F*_o_^2^) + (0.0461*P*)^2^ + 1.6645*P*] where *P* = (*F*_o_^2^ + 2*F*_c_^2^)/3
3522 reflections	(Δ/σ)_max_ = 0.001
252 parameters	Δρ_max_ = 0.29 e Å^−^^3^
7 restraints	Δρ_min_ = −0.23 e Å^−^^3^

### Special details

**Table:**

Geometry. All esds (except the esd in the dihedral angle between two l.s. planes) are estimated using the full covariance matrix. The cell esds are taken into account individually in the estimation of esds in distances, angles and torsion angles; correlations between esds in cell parameters are only used when they are defined by crystal symmetry. An approximate (isotropic) treatment of cell esds is used for estimating esds involving l.s. planes.

### Fractional atomic coordinates and isotropic or equivalent isotropic displacement parameters (Å^2^)

**Table:**

	*x*	*y*	*z*	*U*_iso_*/*U*_eq_	
Li	0.500000	0.4524 (2)	0.250000	0.0204 (6)	
Si	0.57050 (2)	0.24246 (3)	0.21203 (2)	0.01863 (10)	
N1	0.500000	0.30027 (11)	0.250000	0.0182 (3)	
N2	0.37995 (7)	0.54071 (8)	0.16385 (7)	0.0192 (2)	
O8	0.28490 (7)	0.67752 (8)	0.09434 (6)	0.0283 (2)	
C1	0.50526 (11)	0.15288 (11)	0.11638 (9)	0.0283 (3)	
C2	0.66991 (11)	0.15862 (12)	0.29266 (10)	0.0317 (3)	
C3	0.62947 (10)	0.34286 (12)	0.17146 (10)	0.0274 (3)	
C4	0.31661 (9)	0.50169 (10)	0.07406 (8)	0.0238 (3)	
C5	0.37382 (13)	0.44781 (14)	0.03410 (10)	0.0391 (4)	
C6	0.24248 (14)	0.43000 (18)	0.08063 (12)	0.0527 (6)	
C7	0.27067 (11)	0.60331 (12)	0.02589 (9)	0.0307 (3)	
C9	0.35350 (8)	0.63426 (10)	0.16790 (8)	0.0194 (3)	
C10	0.38916 (9)	0.70370 (10)	0.24400 (8)	0.0198 (3)	
C11	0.38259 (10)	0.81222 (10)	0.23259 (9)	0.0255 (3)	
C12	0.41867 (11)	0.87807 (11)	0.30369 (10)	0.0313 (3)	
C13	0.46139 (11)	0.83697 (12)	0.38667 (10)	0.0310 (3)	
C14	0.46664 (10)	0.72887 (11)	0.39881 (9)	0.0280 (3)	
C15	0.43035 (9)	0.66244 (11)	0.32782 (8)	0.0226 (3)	
H1A	0.4564 (12)	0.1901 (13)	0.0681 (10)	0.040 (5)*	
H1B	0.5497 (14)	0.1223 (15)	0.0952 (12)	0.049 (5)*	
H1C	0.4750 (13)	0.0932 (15)	0.1312 (12)	0.041 (5)*	
H2A	0.6467 (14)	0.0989 (15)	0.3147 (12)	0.046 (5)*	
H2B	0.7134 (16)	0.1985 (18)	0.3410 (15)	0.064 (6)*	
H2C	0.7052 (15)	0.1233 (16)	0.2666 (13)	0.054 (6)*	
H3A	0.6706 (14)	0.3083 (15)	0.1489 (12)	0.051 (5)*	
H3B	0.5826 (14)	0.3873 (15)	0.1232 (13)	0.048 (5)*	
H3C	0.6702 (15)	0.3943 (16)	0.2176 (13)	0.052 (5)*	
H5A	0.4240 (14)	0.4948 (15)	0.0334 (12)	0.049 (5)*	
H5B	0.3314 (14)	0.4287 (16)	−0.0254 (14)	0.054 (6)*	
H5C	0.4050 (15)	0.3855 (17)	0.0682 (14)	0.058 (6)*	
H6A	0.2020 (15)	0.4040 (16)	0.0223 (11)	0.064 (6)*	
H6B	0.2736 (14)	0.3696 (14)	0.1122 (13)	0.060 (6)*	
H6C	0.204 (2)	0.465 (2)	0.110 (2)	0.101 (10)*	
H7A	0.3021 (13)	0.6319 (14)	−0.0113 (12)	0.041 (5)*	
H7B	0.2033 (10)	0.5970 (14)	−0.0090 (11)	0.041 (5)*	
H11	0.3552 (12)	0.8399 (12)	0.1757 (9)	0.033 (4)*	
H12	0.4131 (12)	0.9517 (14)	0.2954 (11)	0.038 (5)*	
H13	0.4891 (13)	0.8821 (14)	0.4369 (12)	0.040 (5)*	
H14	0.4949 (11)	0.7004 (12)	0.4566 (9)	0.030 (4)*	
H15	0.4321 (11)	0.5886 (10)	0.3360 (10)	0.026 (4)*	

### Atomic displacement parameters (Å^2^)

**Table:**

	*U*^11^	*U*^22^	*U*^33^	*U*^12^	*U*^13^	*U*^23^
Li	0.0169 (14)	0.0193 (14)	0.0197 (14)	0.000	0.0033 (11)	0.000
Si	0.01801 (18)	0.01873 (17)	0.01628 (17)	0.00254 (12)	0.00498 (13)	−0.00047 (12)
N1	0.0188 (7)	0.0169 (7)	0.0171 (7)	0.000	0.0061 (6)	0.000
N2	0.0158 (5)	0.0211 (5)	0.0162 (5)	−0.0006 (4)	0.0028 (4)	0.0025 (4)
O8	0.0283 (5)	0.0300 (5)	0.0190 (4)	0.0125 (4)	0.0035 (4)	0.0056 (4)
C1	0.0322 (8)	0.0261 (7)	0.0221 (7)	0.0030 (6)	0.0079 (6)	−0.0048 (5)
C2	0.0260 (7)	0.0296 (7)	0.0302 (8)	0.0092 (6)	0.0040 (6)	0.0017 (6)
C3	0.0243 (7)	0.0325 (7)	0.0284 (7)	0.0003 (6)	0.0146 (6)	−0.0001 (6)
C4	0.0207 (6)	0.0236 (6)	0.0170 (6)	−0.0013 (5)	−0.0007 (5)	0.0030 (5)
C5	0.0380 (9)	0.0391 (9)	0.0242 (7)	0.0102 (7)	−0.0007 (7)	−0.0097 (6)
C6	0.0419 (10)	0.0597 (12)	0.0316 (9)	−0.0306 (9)	−0.0064 (8)	0.0109 (8)
C7	0.0322 (8)	0.0314 (7)	0.0196 (6)	0.0088 (6)	0.0034 (6)	0.0033 (5)
C9	0.0143 (6)	0.0238 (6)	0.0183 (6)	0.0030 (5)	0.0057 (5)	0.0061 (5)
C10	0.0168 (6)	0.0234 (6)	0.0208 (6)	0.0043 (5)	0.0096 (5)	0.0028 (5)
C11	0.0271 (7)	0.0250 (7)	0.0246 (7)	0.0072 (5)	0.0116 (6)	0.0062 (5)
C12	0.0388 (8)	0.0220 (7)	0.0345 (8)	0.0056 (6)	0.0176 (7)	0.0010 (6)
C13	0.0351 (8)	0.0325 (7)	0.0277 (7)	0.0027 (6)	0.0159 (6)	−0.0065 (6)
C14	0.0322 (7)	0.0336 (7)	0.0204 (6)	0.0070 (6)	0.0137 (6)	0.0024 (5)
C15	0.0234 (6)	0.0240 (6)	0.0219 (6)	0.0061 (5)	0.0116 (5)	0.0045 (5)

### Geometric parameters (Å, º)

**Table:**

Li—N1	1.942 (3)	C4—C6	1.518 (2)
Li—N2	2.1263 (18)	C4—C7	1.5353 (18)
Li—N2^i^	2.1264 (18)	C5—H5A	0.99 (2)
Li—Si^i^	3.074 (3)	C5—H5B	0.97 (2)
Si—N1	1.6782 (7)	C5—H5C	0.98 (2)
Si—C3	1.8824 (15)	C6—H6A	0.976 (15)
Si—C1	1.8881 (14)	C6—H6B	0.945 (15)
Si—C2	1.8900 (14)	C6—H6C	1.04 (3)
N2—C9	1.2756 (16)	C7—H7A	1.029 (19)
N2—C4	1.5022 (16)	C7—H7B	0.956 (14)
O8—C9	1.3610 (15)	C9—C10	1.4700 (18)
O8—C7	1.4486 (17)	C10—C15	1.3950 (18)
C1—H1A	0.968 (14)	C10—C11	1.3964 (18)
C1—H1B	1.00 (2)	C11—C12	1.381 (2)
C1—H1C	0.988 (19)	C11—H11	0.944 (13)
C2—H2A	0.99 (2)	C12—C13	1.383 (2)
C2—H2B	0.95 (2)	C12—H12	0.949 (18)
C2—H2C	0.96 (2)	C13—C14	1.392 (2)
C3—H3A	0.99 (2)	C13—H13	0.967 (18)
C3—H3B	1.00 (2)	C14—C15	1.3843 (19)
C3—H3C	1.01 (2)	C14—H14	0.963 (13)
C4—C5	1.511 (2)	C15—H15	0.952 (13)
			
N1—Li—N2	122.02 (7)	C5—C4—C7	111.64 (12)
N1—Li—N2^i^	122.02 (7)	C6—C4—C7	111.07 (14)
N2—Li—N2^i^	115.95 (14)	C4—C5—H5A	111.0 (11)
N1—Li—Si^i^	29.36 (3)	C4—C5—H5B	109.1 (12)
N2—Li—Si^i^	108.77 (5)	H5A—C5—H5B	108.4 (16)
N2^i^—Li—Si^i^	127.05 (6)	C4—C5—H5C	109.6 (12)
N1—Si—C3	110.90 (7)	H5A—C5—H5C	108.2 (16)
N1—Si—C1	114.23 (6)	H5B—C5—H5C	110.6 (16)
C3—Si—C1	104.72 (7)	C4—C6—H6A	107.3 (13)
N1—Si—C2	115.55 (6)	C4—C6—H6B	108.6 (13)
C3—Si—C2	106.22 (7)	H6A—C6—H6B	104.3 (17)
C1—Si—C2	104.31 (7)	C4—C6—H6C	113.5 (16)
Si—N1—Si^i^	127.83 (9)	H6A—C6—H6C	113 (2)
Si—N1—Li	116.09 (4)	H6B—C6—H6C	109 (2)
Si^i^—N1—Li	116.09 (4)	O8—C7—C4	104.34 (10)
C9—N2—C4	106.52 (10)	O8—C7—H7A	109.1 (10)
C9—N2—Li	131.72 (11)	C4—C7—H7A	113.4 (10)
C4—N2—Li	121.09 (9)	O8—C7—H7B	107.2 (11)
C9—O8—C7	105.29 (10)	C4—C7—H7B	112.9 (11)
Si—C1—H1A	111.5 (11)	H7A—C7—H7B	109.6 (15)
Si—C1—H1B	111.1 (11)	N2—C9—O8	118.04 (11)
H1A—C1—H1B	107.0 (15)	N2—C9—C10	127.49 (11)
Si—C1—H1C	112.2 (10)	O8—C9—C10	114.47 (11)
H1A—C1—H1C	108.4 (15)	C15—C10—C11	119.42 (12)
H1B—C1—H1C	106.4 (15)	C15—C10—C9	120.72 (11)
Si—C2—H2A	113.3 (11)	C11—C10—C9	119.86 (11)
Si—C2—H2B	111.9 (13)	C12—C11—C10	120.24 (13)
H2A—C2—H2B	108.5 (17)	C12—C11—H11	120.5 (10)
Si—C2—H2C	112.2 (12)	C10—C11—H11	119.2 (10)
H2A—C2—H2C	101.5 (16)	C11—C12—C13	120.21 (13)
H2B—C2—H2C	108.8 (18)	C11—C12—H12	119.8 (11)
Si—C3—H3A	110.5 (11)	C13—C12—H12	120.0 (11)
Si—C3—H3B	113.2 (11)	C12—C13—C14	120.00 (14)
H3A—C3—H3B	106.9 (15)	C12—C13—H13	121.0 (11)
Si—C3—H3C	113.2 (11)	C14—C13—H13	118.9 (11)
H3A—C3—H3C	107.8 (16)	C15—C14—C13	120.07 (13)
H3B—C3—H3C	104.9 (15)	C15—C14—H14	120.0 (10)
N2—C4—C5	111.37 (11)	C13—C14—H14	119.9 (10)
N2—C4—C6	107.51 (12)	C14—C15—C10	120.03 (12)
C5—C4—C6	112.48 (16)	C14—C15—H15	120.2 (9)
N2—C4—C7	102.23 (10)	C10—C15—H15	119.8 (9)
			
C3—Si—N1—Si^i^	176.77 (5)	Li—N2—C9—O8	165.63 (9)
C1—Si—N1—Si^i^	58.73 (6)	C4—N2—C9—C10	175.66 (12)
C2—Si—N1—Si^i^	−62.30 (6)	Li—N2—C9—C10	−13.9 (2)
C3—Si—N1—Li	−3.23 (5)	C7—O8—C9—N2	−7.87 (16)
C1—Si—N1—Li	−121.27 (6)	C7—O8—C9—C10	171.71 (11)
C2—Si—N1—Li	117.70 (6)	N2—C9—C10—C15	−24.5 (2)
C9—N2—C4—C5	133.79 (13)	O8—C9—C10—C15	155.95 (12)
Li—N2—C4—C5	−37.89 (15)	N2—C9—C10—C11	154.97 (13)
C9—N2—C4—C6	−102.56 (15)	O8—C9—C10—C11	−24.56 (17)
Li—N2—C4—C6	85.76 (15)	C15—C10—C11—C12	1.6 (2)
C9—N2—C4—C7	14.45 (14)	C9—C10—C11—C12	−177.93 (13)
Li—N2—C4—C7	−157.23 (10)	C10—C11—C12—C13	−0.1 (2)
C9—O8—C7—C4	16.31 (14)	C11—C12—C13—C14	−1.1 (2)
N2—C4—C7—O8	−18.50 (14)	C12—C13—C14—C15	0.9 (2)
C5—C4—C7—O8	−137.66 (13)	C13—C14—C15—C10	0.5 (2)
C6—C4—C7—O8	95.91 (15)	C11—C10—C15—C14	−1.77 (19)
C4—N2—C9—O8	−4.81 (15)	C9—C10—C15—C14	177.72 (12)

Symmetry code: (i) −*x*+1, *y*, −*z*+1/2.
